# The integrated reflective feedback tool: Experiential learning in resource limited settings

**DOI:** 10.4102/phcfm.v16i1.4394

**Published:** 2024-11-07

**Authors:** Liesl Visser, Nazlie Beckett

**Affiliations:** 1 Department of Family Medicine, College of Medicine, Mohammed Bin Rashid University, Dubai, United Arab Emirates; 2 Department of Family, Community and Emergency Care, Faculty of Health Sciences, University of Cape Town, Cape Town, South Africa

**Keywords:** feedback, reflection, debriefing, limited resources, rural, consultation skills, primary care consultations, family medicine

## Abstract

Combining key elements from the learning tools of reflection, feedback and debriefing into a single reflective event is a novel concept. This powerful combination amalgamates into a tool useful in experiential learning. Linking the three concepts in a way that combines self-reflection in a debriefing framework, powered by trusted peer feedback resulted in a new teaching tool. It was piloted within the clinical setting of the primary health care (PHC) clinics in the Saldanha Bay sub-district in South Africa among final (6th) year undergraduate medical students of the University of Cape Town. This sub-district is a peri-urban community and encompasses a district health care system with less resources in both human and infrastructure than in an urban area. Being intentional and analytical as to optimal use of every resource not only impacts the students but also alleviates the pressure on clinician supervisors of activities. Pairing students alleviates the pressure of the insufficient number of consultation rooms for solo consultations and the pressure on the single supervising clinician when the consultations are conducted simultaneously. Using this tool at the end of the session facilitates the reflection and experiential learning of both the students individually. Self-reports indicated this tool kept both students engaged throughout all consultations and facilitated peer learning Alternating between being the doctor and the observer and knowing what roles entailed, the students never felt victimised in the reflection and feedback on their performance. And individually they developed their self-reflection and providing feedback as life skills.

## Introduction

Reflection, feedback and debriefing are individually valuable tools in the teaching environment and, when purposefully combined, can have an exponential impact. Being intentional in the use and facilitation of this constructivist process has great impact on the student’s experiential learning and their long-term reflective abilities. Utilising this combined method can enhance learning in any clinical setting and is even more impactful in resource-limited settings.

Rural medical training has a smaller pool of both infrastructure and human resources and requires conscientious use of its resources. Both the students and clinician supervisors of these activities are impacted by this. The value of training undergraduates and postgraduates in this setting has been well documented over the past 20 years, benefiting both students and the communities they serve.^1^ Simplifying and enhancing training opportunities in a rural platform emphasises the need to be intentional and analytical about maximising all available resources.

The combination of self-reflection in a debriefing framework as powered by trusted peer feedback resulted in the new teaching tool. It was piloted within the clinical setting of the Saldanha Bay sub-district (SBM). A group of five to seven final (6th) year undergraduate medical students of the University of Cape Town (UCT) spend a compulsory 25% of their Family Medicine rotation in a rural or peri-urban or district health setting.

## Educational innovation

Reflection, feedback and debriefing are individually well-established teaching tools in medical education:

- **Reflection:** In this teaching tool, the student is asked to rethink an interaction and communicate its impact.
- **Feedback:** This involves a formative assessment where the student receives a report back on their level of success in performing a task, mostly in a unidirectional conversation.
- **Debriefing:** It is mostly used in simulation-based teaching and emphasises bidirectional, interactive discussion with emphasis on the student’s experience, comprehension of what happened and its meaning to them.^2^

Overlapping commonalities are evident among these tools as well as their contribution in constructivism and experiential learning. Donald Schön’s model of reflective learning describes reflection-in-action and reflection-on-action as different times of reflection that will elicit different reflections.^3^ Being mindful of the different reflections and their consequences, the idea of an integrated model for clinical practice emerged. The model used for providing feedback is the feedback sandwich model. This requires an observer to tell the person what they did well as the outer buns of the statement and then fill the middle with the filling of what they believe the person could have been done better.^4^ Combining the different aspects of self-reflection in a debriefing framework powered by trusted peer feedback resulted in the new teaching tool that was piloted within the clinical setting of the SBM.

Situated 130 km north of Cape Town on the West Coast of South Africa, SBM was the first site identified by UCT for decentralised, rural or peri-urban training of health and rehabilitation students. The SBM serves an area of more than 2000 km^2^ and population of 123 000 people. Five to seven medical students are placed at this site per allocation, under the direct supervision of a Family Physician (FP) and has access to a 112-bed district hospital (run by 12 fulltime, non-specialised, generalist medical doctors) and eight primary health care (PHC) clinics.^5^

One of the students’ scheduled weekly activities is a primary care outreach at a nurse-led PHC clinic. Other than the reception, waiting area and pharmacy, the clinic has seven consultation or treatment rooms in which the four primary care sisters perform all their duties. There are thus not enough rooms for each of the five to seven students to consult patients individually. The FP thus instructs the students to pair up with their clinical partner for this session. The students then alternatively conduct and observe the consultations, and the FP oversees all the consultations and procedures conducted. The FP oversees the consent from the patients prior to the allocations to the students and then the students also confirm the patient’s consent.

Importantly, prior to the room allocation, the FP explains to the students that integrated reflective debriefing will happen at the end of the outreach session and the value of this. This does not only place it on the agenda but also provides some insights into the learning process and making the students aware of their thought processes. Their changing roles within the consultation room between being the ‘doctor’ and the ‘observer’ in the consultations are also explained as well as what the expectation of each role is.

Student A will conduct consultation 1 while student B observes, focussing on the consultation skills of their peer using the same marking guide as the Family Medicine department uses for summative student assessment. During the next consultation, the roles will swap around as student B becomes the doctor and student A the observer.

At the end of the session, the FP then meets with each of the rooms individually to facilitate the debriefing session. The sequencing and method of this facilitating are significant. It is a structured incorporation of reflection-in-action and reflection-on-action, as per Donald Schön’s reflection model, empowered by peer feedback presented in the feedback sandwich method.

The debriefing style of the tool is facilitated by the clinical supervisor or FP. The tool’s method is first conducted for student A, and thereafter, the method is repeated with the focus on student B and the consultation(s) they had conducted. A very short summary of the patient’s presentation or diagnosis is given to ensure that everyone in the room is talking about the same consultation or patient. The first question to student A is what went through their mind as they were conducting the consultation: this refers to Schön’s reflection-in-action. The second question is still to student A about what went well in the consultation. The third question is addressed to student B about what they observed worked well or what went well in the consultation. The follow-up question is then what they think could perhaps have been done in a slightly different way and why they say that. And the third question to student B is what their overall (short) reflection on the consultation is. The three questions to student B refer to the feedback sandwich method. The attention is now returned to student A with the question about their opinion on their peer’s feedback by asking them what do they think they could perhaps have done differently, and what impact they believed it would have had. And then the last question is asked on what their take-home lesson or reflection is, and this is in keeping with Schön’s reflection in action. See Table 1-A1 for a summarised concise version.

The FP’s role is to guide the conversations and reflections and correct where needed. Yet their experience and insight are invaluable in this setting. Facilitating this conversation prevents both harshness and superficiality.

## Discussion

Allocating two students per consultation room can be seen as suboptimal clinical exposure as it might lead to either both students attempting to be in charge of the consultation or the disengagement of one of the students while the other is conducting the consultation.

This was the dilemma that the FP was facing. In consultation with peers and medical education experts, learning theories, models, concepts and tools were reviewed. None were sufficient to achieve the outcomes set to achieve, namely debriefing student A after the consultation while keeping the student B engaged and furthermore teach the students the lifelong skills of self-reflection and providing feedback. The tool was developed by the FP, piloted and refined to its current format.

Allocating specific tasks to the two students and thus actively engaging both students in each consultation change a single consultation into a dynamic learning experience for both. The students’ feedback reports that they felt safe and engaged during these consultations despite being more than one person in the consultation room while the consultation was conducted. The students also reported that they were never before made aware of their inner conversation that was happening as they were busy with their activities, and how this influences them in this consultation as well as future consultations. They liked receiving the good and bad feedback from their peer as this often exposed blind spots that they themselves were not aware of. They also reported that this mindful engagement facilitated their learning from each other’s consultations. They felt safe with their peer providing feedback as they know each other, and they did not feel that there is a power dynamic in the room, in comparison to a doctor giving feedback. They were appreciative that the process was led and facilitated by the FP. The students themselves had taken this further and repeated the same process, self-facilitated this time, when consulting with patients in the emergency department after hours.

The students felt that this opportunity served as a reminder that giving feedback to someone could have a lifelong impact on others and on yourself as it inspires self-reflection. Ultimately, the facilitated reflection and feedback were more powerful as it engaged both students and required reflection from both. The sequencing of the questions is logical and builds on each other. This debriefing technique enhanced the depth of engagement and learning. Altogether, it constructs and enhances the experiential learning of each individual student.

## Conclusion

When being honest and reflective about challenges that a training site faces, it is possible to look through known teaching tools and find that none of them is sufficiently fitting for the specific challenge. Through some reflection and innovation, a fit-for-purpose tool can be constructed through adaptation and modification of known successful tools that might be partially fitting. The integrated reflective feedback tool is such a creation. The challenges of growing student numbers as well as resource constraints, especially in rural areas, do not have to have a negative impact. It does, however, require a more careful use of resources and shift from the traditional apprenticeship model. Yet, this is a tool that could become the norm when practising skills, especially consultation skills, in more senior clinical students.

Not having enough consultation rooms for solo consultations or enough clinical supervisors to observe all consultations individually, does not have to be seen as a drawback on a training site. Having two students per consultation room is beneficial and stimulates growth and development in other ways than conducting every consultation in solo. Purposeful, non-vindicative peer feedback can expose a blind spot, which can be remediated by self-reflection long after an interaction. And with this conversation being guided by a clinical supervisor, it provides structure, clinical expertise as well as safe space for feedback and reflection. And once being comfortable and acquainted with the tool, it can be self-applied as the students demonstrated when using it during their emergency department shifts.

Beyond these specific occasions where the focus was on consultation skills, the explicitness of this new tool further coaches these students in using reflection and independent judgement for the future: to become problem solvers and acknowledge the learning potential of all their clinical experiences.

The authors would like to thank the Faculty of Health Sciences at the University of Cape Town.

## Appendix 1

**TABLE 1-A1:** Integrated reflective feedback tool.

Question Nr	To which student	Question wording	What part of models as reference
1	A	What went through your mind as you were conducting the consultation?	Schön: Reflection-in-action
2	A	What went well in your consultation?	(Reflection)
3	B	What did you observe that went well?	Feedback sandwich
4	B	What do you think could perhaps have been done in a slightly different way, and why do you say that?	Feedback sandwich
5	B	What is your overall (short) reflection on the consultation?	Feedback sandwich
6	A	What do you think you could perhaps have done a bit differently and what impact do you think it would have had?	(Reflection)
7	A	What is your take home message for yourself?	Schön: Reflection-on-action
